# BE-WEL trial (breast: evaluation of weight and exercise for lymphoedema) testing weight control and exercise programmes for women with breast cancer related lymphoedema: a feasibility trial

**DOI:** 10.1007/s10549-024-07356-0

**Published:** 2024-05-17

**Authors:** Michelle Harvie, Karen Livingstone, Debbie McMulllan, Mary Pegington, Cheryl Lombardelli, Judith Adams, Maggie Farragher, Emma Barrett, Nigel Bundred

**Affiliations:** 1 The Nightingale and Prevent Breast Cancer Centre, Manchester University Hospital Foundation NHS Trust, Manchester, UK; 2 Division of Cancer Sciences, School of Medical Sciences, Faculty of Biology, Medicine and Health, The University of Manchester, Wilmslow Road, Manchester, M20 4BX UK; 3 Manchester Academic Health Science Centre and Radiology, Central Manchester University Hospitals NHS Foundation Trust and University of Manchester, Manchester Royal Infirmary, Manchester, UK; 4 Trafford Community, Manchester University Hospital Foundation NHS Trust, Manchester, UK; 5 Research and Innovation, Manchester University Hospital Foundation NHS Trust, Manchester, UK

**Keywords:** Breast cancer related lymphoedema, Weight loss, Obesity, Resistance exercise, Perometer

## Abstract

**Purpose:**

A combined body weight loss and upper body/arm exercise programme is a potential strategy for managing Breast cancer related lymphoedema (BCRL), but there is limited data on the best method for delivery or its potential efficacy.

**Methods:**

Fifty-seven women with overweight/obesity and BCRL were randomised to a 12 week supervised (*n* = 12) or home-based combined weight loss and upper body/arm exercise programme (*n* = 16), a home-based upper-body arm exercise only programme (*n* = 17) or standard care (*n* = 12). Primary outcomes were uptake, retention and changes in weight and change in Relative Arm Volume Increase (RAVI) using analysis of covariance (ANCOVA).

**Results:**

Sixteen percent of women invited joined the study and 49 completed the trial (85% retention). Reductions in weight occurred in the supervised and home-based weight control and exercise programmes; Mean (95% CI) change compared to standard care − 1.68 (− 4.36 to − 1.00), − 2.47(− 4.99 to − 0.04) Kg. Reductions in perometer assessed RAVI were seen in the supervised and home-based combined weight control and arm exercise groups and the weight stable home-based arm exercise only group: mean (95% CI) change compared to standard care − 2.4 (− 5.0 to + 0.4),− 1.8 (− 4.3 to + 0.7), − 2.5(− 4.9 to − 0.05)%.

**Conclusion:**

Women with BCRL and overweight and obesity engaged in diet and exercise weight loss programmes. Both weight loss/arm exercise programmes led to modest changes in weight and BCRL. Comparable reductions in BCRL were reported in the weight stable group undertaking arm exercise only. The independent and combined effects of weight loss and exercise on BCRL need further study.

**Trial registration:**

ISRCTN86789850 https://doi.org/10.1186/ISRCTN86789850, registered 2011.

**Supplementary Information:**

The online version contains supplementary material available at 10.1007/s10549-024-07356-0.

## Introduction

Breast cancer related lymphoedema (BCRL, gross swelling of the arm) is a chronic distressing condition affecting 20% of breast cancer patients with few effective treatments [1–3]. BCRL has multidimensional physical and psychosocial morbidities. Patients report the limb heavy and painful, experiencing impaired limb function and reduced shoulder mobility. BCRL markedly reduces quality of life (QOL) [3]. Approximately 70% of women who develop BCRL are living with overweight or obesity [3]. Weight control is included in many BCRL practice guidelines however these are mainly based on consensus since there are few data on the effects of weight loss for reducing arm swelling, improving QOL and function in patients with breast cancer related BCRL [4].

Weight loss may reduce excess arm volume through anti-inflammatory effects, by reducing subcutaneous fat between lymph vessels, reducing circulating dietary fat in lymphatic channels or increased effectiveness of compression garments. Some weight loss studies have shown reduced excess arm volume [5–7], whilst others have shown reductions in volume of the affected and unaffected arm but no difference in the inter-limb volume difference [8]. Recent studies have shown upper-body resistance exercise reduces arm volume and lymphoedema flare-ups and symptoms, possibly due to increased muscle function and vascular flow [9, 10]. A combined body weight loss and upper body/arm exercise is a potential effective management for BCRL but there is limited data on its potential for managing BCRL, whether this strategy is superior to upper-body resistance exercise alone, or the most effective way that this could be delivered.

This paper reports findings from a feasibility pilot trial to assess the potential benefits of either a supervised or home-based combined weight loss and upper body/arm exercise programme or a home-based upper-body arm exercise only programme compared to standard care for women with overweight/obesity and BCRL.

The primary aims of this trial were to assess the following in the three different programmes and the standard care group:

1. Uptake and retention to the trial.
2. Engagement to the three programmes (classes attended/calls received in the 12-week programmes).
3. Changes in body weight and total body fat and fat free mass (FFM) assessed with dual-energy X-ray absorptiometry (DXA) between baseline and 12 weeks.
4. Changes in arm swelling between baseline and 12 weeks measured by perometer assessed Relative Arm Volume Increase (RAVI).

Secondary aims

1. Adherence to the diet and physical activity recommendations evidenced by self-reported dietary intake and physical activity.
2. Self-reported symptoms assessed with the Functional Assessment of Cancer Therapy (FACT scales).
3. The correlation between changes in lymphoedema assessed with bioelectrical impedance and RAVI over the 12-week programme to inform best measurement methods for evaluating changes in BCRL in weight loss/resistance exercise programmes.

## Method

### Trial design

**Fig. 1:**
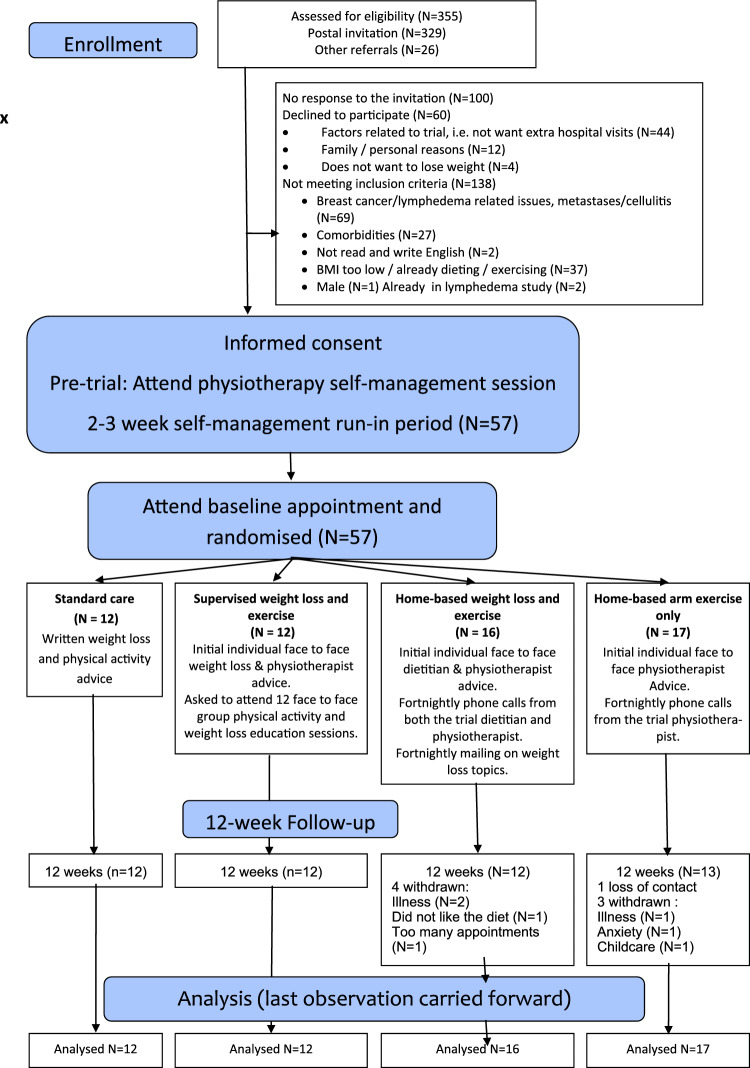
Consolidated Standards of Reporting Trials (CONSORT) for the BEWEL Trial

A randomised four-arm feasibility trial was undertaken amongst patients in a BCRL clinic at Manchester University Hospital Foundation NHS Trust (MFT). The trial had a pragmatic design and aimed to recruit 15 participants to each of the four groups following previously published rule of thumb recommendations for feasibility studies [11, 12]. The trial included women who were receiving maintenance therapy for breast cancer related BCRL (i.e. compression sleeves and/or manual lymphatic drainage) and with overweight/obesity; body mass index (BMI) greater than 25 kg/m^2^. Patients were invited to the trial either face to face in clinic or via an invite letter and a follow up phone call to determine interest in the trial. Their BCRL had to have been stable over the previous three months defined as no intensive therapy i.e. no manual decongestive treatment, no recorded 10% change in volume of the affected arm lasting > 7 days (assessed with perometer), no lymphoedema related infections (cellulitis) requiring antibiotics. They also needed to understand written instructions. Full eligibility criteria are reported in Supplementary Table 1. Patients gave written informed consent, and the research was approved by the NRES Committee North-West—Greater Manchester North Research Ethics Committee (11/H1011/2). At recruitment participants were asked to attend a class to reinforce optimum self-management of BCRL run by the trial BCRL specialist physiotherapist. This class reinforced optimum skincare, arm mobility exercises, simple lymphatic drainage (SLD) and use of compression hosiery particularly when exercising. Patients were asked to re-attend 2–3 weeks later for baseline assessment and randomisation to one of four intervention groups. Measurements were assessed at recruitment (pre-trial), baseline (after 2–3 week run in) and after 12-weeks follow up in the 4 groups (Fig. 1).

### Randomisation

Patient randomisation was undertaken using a sealed envelope method. An independent statistician made random allocation cards using computer-generated random numbers which were placed in groups of sealed opaque envelopes according to three stratification factors. (1) The presence of mild (< 20% excess) or moderate severe (> 20% excess arm volume) BCRL [13]. (2) Length of time with chronic BCRL (> or < 1 year). (3) Previous axillary radiotherapy/no axillary radiotherapy.

The researchers opened the envelopes to allocate patients to a treatment group 1.

### Interventions

1. The standard care (control) group were encouraged to continue with the optimal self-management described above. They also received comprehensive written information on the importance of weight control, how to follow a healthy Mediterranean diet and to undertake 150 min of moderate intensity physical activity/week. This group were offered individualised diet and physical activity advice from the trial dietitian and physiotherapist at the end of the trial.
2. The supervised weight control and upper body/arm exercise group entered a 12-week combined weight control (diet and cardiovascular exercise) and upper-body/arm exercise programme. Participants received initial individualised diet advice from the trial dietitian to follow a daily 25% calorie restricted Mediterranean diet below their estimated energy requirements and to increase cardiovascular exercise to 5 episodes of 30 min exercise per week as described previously [14]. The weight loss programme aimed to achieve a 5% weight loss (~ 15% reduction in body fat) over the trial period. The Mediterranean diet provides 30% energy from fat (15% monounsaturated, 8% polyunsaturated, 7% saturated), 25% energy from protein and 45% from wholegrain carbohydrate. The trial physiotherapist advised patients to undertake progressive 12-week upper body/arm exercise programme aimed to improve mobility and strength of the affected arm, shoulder, thoracic and cervical spine. Exercises aimed to correct and improve posture and to facilitate increased functional ability of the upper limb. The programme utilised established behavioural techniques for weight control, i.e. self-monitoring of food and exercise with diaries and goal setting [15].
  Participants were invited to attend 12-weekly group exercise and diet education classes at the gym facility at MFT. The physiotherapy-led sessions comprised a warm-up, 20–30 min cardiovascular exercise (50–80% age-adjusted heart rate maximum) and the progressive 12-week upper body/arm exercises to improve arm mobility, strength and improve posture. Participants were also asked to complete the resistance exercise at least 2 additional times/each week at home. The diet and behaviour change educational component was based on the trans-theoretical model of behaviour change and was delivered by the trial dietitian and covered topics including healthy cooking, motivation, problem solving, and body image.
3. The home-based weight control and upper body/arm exercise group received a 12-week home-based weight control (diet, cardiovascular exercise) and upper-body arm exercise programme. This group received initial individualised diet and cardiovascular exercise from the trial dietitian and physiotherapist as described above. The physiotherapist also demonstrated the exercises for the upper body/arm exercise programme which they were asked to undertake at least 3 times per week and provided a booklet for the progressive 12-week arm exercise programme. Individual diet and exercise goals and recommendations were reinforced with fortnightly phone calls from the trial dietitian to check adherence and address individual problems. Subjects were mailed a summary of key motivational, behavioural, diet and exercise issues after each phone call. This group also received six fortnightly mailings which included identical diet, exercise and weight control information received by the supervised group during their supervised education session.
4. Home-based upper-body arm exercise only programme. This group received identical instruction on the progressive 12-week arm exercise programme as the home-based group and fortnightly phone calls from the trial physiotherapist.

### Primary outcome measures

1. Uptake and retention to the study

The number of women who were invited and consented to join the trial and who were not interested or not eligible to take part were recorded. Also, retention to the trial at 12-weeks in each of the groups aiming to achieve an uptake of 25% amongst those who were eligible for the study, and retention of 80% in the trial period.

2. Engagement to the programmes

Engagement to the programmes was assessed by attendance to classes (*n*) and receipt of scheduled phone calls (*n*).

3. Changes in body weight, body fat and FFM

Weight was assessed using a segmental multi-frequency body composition monitor (Tanita MC-180MA scale, Tanita Europe, Amsterdam, The Netherlands) and body fat and FFM were estimated from supine DXA using Hologic A Discovery software (Hologic Inc. Marlborough, MA, USA) and corrected for unilateral artefacts, i.e. metallic implants and BCRL, as described previously [14].

4. Changes in arm swelling (compared to contralateral arm)

This was assessed using a perometer as described previously [16]. Briefly a mean of two arm measurements at each visit using a 350S perometer with standard software (Pero System, Germany) to reduce intra and inter operator variation. RAVI was calculated using the formula which allows for changes in the contralateral limb reducing the influence of arm dominance: (A2 − U2/U2) × 100 − (A1 − U1/U1) × 100 where A2 = volume of the treated arm post intervention, U2 = baseline volume for treated arm, A1 = volume of the contralateral arm post intervention and U1 = baseline volume of the contralateral arm.

### Secondary objectives

1. Changes in self-reported dietary intake of energy, total fat, saturated fat, protein, carbohydrate (7-day food diaries analysed with Wisp version 3 (Tinuviel Software, Anglesey, Wales) and the amounts of moderate or vigorous intensity cardiovascular physical activity exercise (7- day activity diary) within each of the groups.
2. Self-reported symptoms were assessed with the Functional Assessment of Cancer Therapy-Breast + 4 (FACT-B + 4) which included sub scales for physical, functional and fatigue, breast cancer specific issues and arm function. FACT-B trial outcome indicator ((TOI) sum of physical, functional and breast cancer specific sub scales), FACT-F TOI (sum of physical, functional and fatigue sub scales), FACT Arm subscale and perception of arm swelling (question B3 in the breast cancer specific sub scale on the FACT questionnaire) were reported [17].
3. Changes in arm swelling using multi-frequency bio impedance electrical analysis (BEA) (L-Dex U400 bio impedance spectroscopy device) to assess if this is a useful alternative method to the perometer for assessing changes in BCRL with weight loss/upper body resistance exercise [16].

### Data analysis

Categorical variables, including primary outcomes ‘uptake’ and ‘retention’, are reported as frequencies and percentages. Continuous variables are reported as mean and 95% confidence interval median (interquartile range). Changes in primary outcomes ‘weight’, ‘excess arm volume’ (RAVI) and ‘body composition’ are summarised by group, and differences between groups, intervention vs control, were assessed using analysis of covariance (ANCOVA) with baseline measures included as covariates. Last observation carried forward (LOCF) was used to impute missing data. All primary and secondary endpoints are reported descriptively only, with no formal testing of differences between the groups.

The correlation of the two methods for assessing changes in lymphoedema from baseline to end of study, the ratio of impedances between limbs (BEA) and inter-limb volume differences (perometer), are reported using Kendall’s rank-based tau coefficient. All analyses are two-sided using the 5% significance level and were conducted using statistical software R version 4.2.1.

## Results

### Primary outcomes

1. Uptake and retention

Fifty-seven women with stable lymphoedema were recruited to the trial and were randomised to the four arms (12 standard care: 12 supervised weight loss and exercise: 16 home-based weight loss and exercise: 17 home-based arm exercise). Uptake and retention is reported in the Consolidated Standards of Reporting Trials (CONSORT) Fig. 1 this represented 16% of those invited (57/355). One hundred and ninety-five patients expressed an interest in joining the study after receiving the patient information sheet (55% of those invited), however 138 (70%) of these patients were not eligible mainly related to breast cancer treatment and lymphoedema related issues. Forty-nine participants completed the trial (85% retention) out of the women from the home-based weight loss and exercise group (25%) and four from the home-based arm exercise only group withdrew or had loss of contact (24%) from the trial before the 12-week review. Baseline characteristics of the patients are reported in Table 1. For the overall group mean (SD) age was 62.6 (8.8) years and BMI 29.8 (4.5) Kg/m^2^. The majority had mild lymphoedema (10–20% RAVI) 82%, whilst 18% had moderate severe lymphoedema (> 20% RAVI). Time since diagnosis with lymphoedema was median (range) 4.0 (0.5–16) years.

2. Engagement with the programmes

**Table 1:** Baseline characteristics of the four groups

	Overall study population (n = 57)	Standard care (*n* = 12)	Supervised weight loss and exercise (*n* = 12)	Home-based weight loss and exercise (*n* = 16)	Home-based arm exercise only (*n* = 17)
Age^a^	62.6 (8.8)	63.8 (6.2)	63.0 (8.9)	61.9 (9.4)	58.2 (9.2)
BMI* (kg/m^2^)^a^	29.8 (4.5)	28.9 (4.2)	30.3 (4.5)	30.08 (5.0)	29.5 (3.5)
% body fat (DXA)^a^	42.9 (4.3)	38.1 (4.0)	37.6 (5.0)	38.0(6.1)	36.8 (3.5)
Post-menopausal^b^	54 (95%)	12 (100%)	12 (100%)	15 (94%)	15 (88%)
Pre/peri-menopausal	3 (5%)	0 (0%)	0 (0%)	1 (6%)	2 (12%)
Previous breast and axillary surgery^b^					
Mastectomy and ANC*	25(44%)	7(59%)	3(25%)	9(56%)	6(35%)
WLE & ANC*	27 (47%)	4 (33%)	9(75%)	6 (38%)	8 (47%)
Mastectomy and SNB*	3 (5%)	1 (8%)	0 (0%)	1 (6%)	1 (6%)
WLE & SNB*	2(4%)	0 (0%)	0 (0%)	0 (0%)	2(12%)
ER + ^b^	46 (81%)	10 (84%)	11 (92%)	13 (81%)	12 (71%)
ER–	11 (19%)	2 (17%)	1(8%)	3(19%)	5(29%)
Previous chemotherapy^b^	28 (49%)	6 (50%)	7 (58%)	9 (56%)	6 (35%)
Previous radiotherapy^b^	37 (65%)	7 (58%)	10 (83%)	9 (56%)	11 (65%)
Previous/current endocrine therapy^b^	46 (80%)	10 (83%)	11 (92%)	13 (81%)	12 (71%)
Number of lymph nodes removed^c^	18 (0–64)	17 (0–18)	22 (4–29)	17 (3–27)	17 (2–64)
Duration of lymphoedema (years)^c^	4.0 (0.5 -16.0)	4.0 (2.5–16.2)	4.0 (0.8–17.0)	2.5 (0.5–16.0)	4.0 (0.5–8.0)
Severity of lymphoedema (% RAVI)^b^					
Mild < 20%	47 (82%)	10 (83%)	10 (83%)	14 (88%)	13 (77%)
Moderate/severe > 20%	10 (18%)	2 (17%)	2 (17%)	2 (12%)	4 (23%)
Time since breast cancer diagnosis (years)^c^	5.0 (0.99–30.0)	5.4 (2.5–16.2)	5.0 (1.3–12.9)	6.8 (1.3–30.3)	4.8 (1.0–20.0)
Ethnicity^b^					
White	51(90%)	12(100%)	10 (83%)	15 (94%)	14(82%)
Black	1 (2%)	0 (0%)	1 (8%)	0 (0%)	0 (0%)
Asian	4 (7%)	0 (0%)	1 (8%)	1 (6%)	2(12%)
Mixed	1 (2%)	0 (0%)	0 (0%)	0 (0%)	1(6%)
Social circumstances					
Married or cohabiting^b^	35 (61%)	8(67%)	9(75%)	9(57%)	9(52%)
Index of multiple deprivation					
Greater Manchester Quintile^b^					
1 (least deprived)	13 (23%)	3 (25%)	2 (17%)	4 (25%)	4 (24%)
2	13 (23%)	1 (8%)	0 (0%)	7 (44%)	5 (29%)
3	14 (25%)	3 (25%)	5 (42%)	2 (13%)	4 (24%)
4	9 (16%)	3 (25%)	2 (17%)	2 (13%)	2 (12%)
5 (most deprived)	8 (14%)	2 (17%)	3 (25%)	1 (12%)	2 (12%)
Co-morbidities^b^					
Respiratory, e.g. asthma, COPD*	11 (19%)	3 (27%)	3 (25%)	3 (21%)	2 (15%)
Psychiatric, e.g. anxiety, depression	6 (11%)	2 (17%)	2 (17%)	0 (0%)	2(12%)
Previous CVD/BP high cholesterol	29 (51%)	8 (67%)	6 (50%)	9 (56%)	6(35%)
Musculoskeletal problems	32 (56%)	8 (73%)	6 (50%)	9 (64%)	9 (68%)
Type 2 diabetes	2(4%)	1(8%)	0 (0%)	1 (6%)	0 (0%)

*ANC axillary node clearance, *WLE *wide local excision, *SNB *sentinel node biopsy, *BMI *body mass index, *COPD *chronic obstructive airway disease, *RAVI* relative arm volume increase, *CVD* cardiovascular disease

^a^Mean (SD)

^b^*n* (%)

^c^Median (range)

Median (interquartile range) class attendance to the 12 supervised weight loss and exercise classes was 9 (5–12) classes. Median (interquartile range) receipt of the 6 scheduled phone calls was 5 (3–5) calls for the home-based weight loss and exercise group and 4 (3–5) calls for the home-based arm exercise only group. These estimates were based on intention to treat so that classes or calls missed due to withdrawal from the programme or loss of contact were included as missed.

3. Change in weight and body composition

The supervised and home-based weight loss and exercise groups both reduced body weight and body fat compared to the control group, but not the arm exercise only group. Twelve-week weight change in supervised weight loss and exercise group − 1.68 (− 4.36 to − 1.00) Kg, home-based weight loss and exercise group − 2.47 (− 4.99 to − 0.04) Kg, home− based arm exercise only group 0.37 (− 2.11 to + 2.85) Kg compared to the standard care group (Table 2).

4. Changes in BCRL measurements

**Table 2:** Changes in weight body composition and relative arm volume increase (perometer) between pretrial, baseline and the end of the 12-week interventions

Measures pre-trial at baseline and 12 weeks					Group difference for changes between baseline & 12 weeks^b^		
	Standard care (*n* = 12)	Supervised weight loss and exercise (*n* = 12)	Home-based weight loss and exercise (*n* = 16)	Home-based arm exercise only (*n* = 17)	Supervised weight loss and exercise vs standard care	Home-based weight loss and exercise vs standard care	Home-based arm exercise only vs standard care
Weight (kg)							
Pretrial	77.45 (69.06–85.83)	79.37 (70.22–88.51)	80.64 (71.05–90.23)	73.85 (68.86–78.84)			
Baseline	77.15 (68.91–85.40)	79.09 (69.74–88.44)	80.83 (71.29–90.38)	74.00 (68.92–79.08)	0.01 (– 1.05 to + 1.08)	0.49 (– 0.51 to + 1.48)	0.44 (– 0.54 to + 1.43)
12 weeks	76.38 (68.42–84.34)	76.59 (67.58–85.61)	77.51 (67.92–87.10)	73.67 (68.45–78.88)	– 1.68 (– 4.36 to – 1.00)	– 2.47 (– 4.99 to – 0.04)	0.37 (– 2.11 to + 2.85)
Body fat (DXA) (kg)							
Baseline	27.1 (23.9–30.4)	30.2 (27.1–33.3)	33.9 (28.4–39.4)	29.0 (25.9–32.1)			
12 weeks	26.6 (23.3–30.0)	28.7 (25.0–32.3)	32.6 (27.5–37.7)	28.6 (25.3–31.9)	– 0.95 (– 2.99 to – 1.09)	– 0.76 (– 2.73 to – 1.20)	0.17 (– 1.71 to + 2.05)
Fat free mass (DXA)(kg)							
Baseline	41.7 (38.4–45.0)	37.0 (33.4–40.6)	43.9 (39.6–48.2)	37.2 (34.2–40.2)			
12 weeks	40.5 (37.3–43.8)	36.6 (33.0–40.1)	42.3 (38.4–46.1)	36.8 (33.7–40.0)	0.78 (– 0.66 to + 2.22)	– 0.39 (– 1.78 to + 0.99)	0.85 (– 0.48 to + 2.18)
Relative arm volume increase (%)							
Pretrial	11.1 (7.2–15.1)	8.7 (4.2–13.3)	13.8 (10.2–17.4)	11.3 (6.2–16.4)			
Baseline	9.8 (5.7–13.9)	9.8 (5.0–15.0)	14.0 (10.1–17.9)	11.3 (6.5–16)	2.3 (– 0.4 to + 5.0)	1.6 (– 0.8 to + 4.0)	1.3 (– 1.1 to + 3.6)
12 weeks	11.2 (6.5–16.0)	8.8 (14.4–13.2)	13.6 (9.5–18.0)	10.2 (5.0–15.3)	– 2.4 (– 5.0 to + 0.4)	– 1.8 (– 4.3 to 0.7)	– 2.5 (– 4.9 to – 0.05)

Full data: standard care group *N* = 12, Supervised weight loss and exercise group *N* = 12, Home-based weight loss and exercise group *N* = 16, home-based arm exercise only Group *N* = 17. Last observation carried forward (LOCF) was used to impute missing data

^a^Mean (95% CI) for values at pre-trial, baseline and 12 weeks

^b^Mean (95% CI) for change from pre- trial to baseline and change from baseline to 12 weeks versus the standard care group from pairwise comparisons

Compared to the standard care group there were small reductions in relative arm volume in the supervised weight loss and exercise group. Mean (95% CI) difference − 2.4 (− 5.0 to + 0.4), and the home− based weight loss and exercise programme − 1.8 (− 4.3 to 0.7) and the exercise group –2.5 (− 4.9 to − 0.05) (Table 2).

### Secondary outcomes

1. Changes in self-reported dietary intake and physical activity

The home-based weight loss and exercise group reported the largest reductions in energy, fat and carbohydrate aligning with the greater weight reduction in this group. There was a range of changes in self-reported minutes of moderate and vigorous physical activity between baseline and 12 weeks in each of the groups; mean (95% CI) standard care – 194 (− 370 to − 17), supervised weight loss and exercise − 201 (− 482 to –81), home− based weight loss and exercise + 16 (− 207 to + 239) and the arm exercise only group − 166 (− 428 to + 95) (Supplementary Table 2).

2. Self-reported symptoms

All groups reported increased scores for FACT-B TOI and FACT-F TOI indicating improved quality of life, with the exception of the supervised weight loss and exercise group. There were no changes in the arm sub scale or self-rated arm swelling in any of the groups (Supplementary Table 3).

3. Changes in arm swelling using BEA

**Fig. 2:**
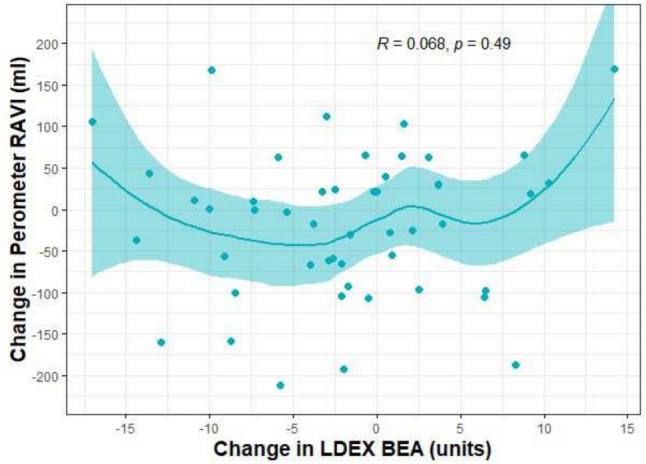
Relationship between change in LDEX BEA and RAVI assessed with a perometer measurements using Kendall Correlation Analysis across all trial participants

There were large variable changes in BEA at the end of the 12-week study, but little change in perometer measures. There was no correlation between changes in the perometer (ml) and the BEA measurements (%): Kendall’s correlation *R* = 0.068 (95% CI − 0.157 to 0.274, *P* = 0.49) (Fig. 2).

## Discussion

We have shown it is feasible to recruit and engage women with breast cancer to a trial testing combined dietary weight loss and upper-body resistance exercise programmes. Reductions in weight and body fat occurred in the supervised and home-based weight control and exercise programmes. Small reductions in perometer assessed BCRL were seen in the two combined weight control and resistance exercise groups as well as the home-based arm exercise only group. There were large variable changes in BEA at the end of the 12-week study, but little change in perometer measures.

Over half of those invited (mainly through postal invitations) expressed an interest in joining the study suggesting an interest in bespoke diet and exercise weight control programmes in this population. Impersonal mail shot postal invitations are known to have a poor response. Face to face invitations in clinic would enhance these rates and should be considered in future studies [18].

Overall there was a good retention to the trial. Drop-outs occurred in the two home-based groups which may have been due to chance with small numbers or reflect a lower engagement with the trial with these remotely delivered programmes. This contrasts with previous reviews of diet and exercise studies in breast cancer patients where hybrid/remote interventions had better retention than in person only delivery [19, 20].

The diet and exercise intervention groups achieved modest weight loss which are comparable with weight loss trials in breast cancer patients [21]. This appeared to occur alongside overall reductions in energy intake in the groups, rather than measurable increases in energy expenditure, as there were both increases and decreases in self-reported moderate or vigorous physical activity in these groups.

The significance of the 1.9–2.5% reductions in RAVI with the interventions vs control is unclear. Some previous studies have defined the minimal clinically important difference in Ravi at 2.5–3%, however there is no consensus an consistency of the definition of this [22]. The changes in the study herein exceed the reported coefficient of variation on repeat perometer measurements (0.5%) [23]. However they could reflect fluctuations in measurements since similar magnitude changes were seen in these groups after the pre-trial run in period when participants were asked to adhere to standard conservative management. This notwithstanding the comparable reductions in perometer assessed BCRL in the combined weight loss and resistance exercise and the resistance exercise only groups suggest weight loss achieved with our test programmes (~ 4%) were not adding to any effects of resistance exercise on BCRL. A lack of effect of a greater long term weight loss (8% at 12-months) on BCRL was previously reported in the WISER study [8], although positive effects have been reported in other studies with smaller weight loss (2–3% at 12 weeks) [5–7]. The WISER study did not report the benefits of a home-based resistance exercise programme on BCRL as reported here [8]. Different outcomes between studies may reflect different trial populations and different methods of measurement of BCRL. The WISER population had a longer duration of BCRL (8 vs. 4 years) and higher levels of obesity than our study and the previously reported weight loss studies [5–7] (median BMI 34 vs. 30 kg/m^2^). These factors combined may reduce the impact of weight loss and exercise on BCRL. The WISER study reported change in BCRL with a perometer, whilst the other studies reported water displacement [7] and circumference measurements [5, 6].

We included self-report measures of quality of life and arm swelling and function which are considered to be clinically meaningful outcomes alongside objective measures of lymphoedema [22]. Modest improvements in all groups with the exception of the supervised diet and exercise group is likely to be a chance finding with the small sample size and we are unable to draw any conclusions from this result. A recent study associated a 29% reduction in volume of oedema with a 25% change in Lymphoedema Life Impact Scale. The large effects in this study may be associated with the larger reduction in RAVI in this study or with the choice of scale [7].

Perometer assessed RAVI is an accepted objective measure of BCRL along with BEA and subjective measurements of patient reported symptom outcomes [24]. The changes in perometer RAVI in the current study were not correlated with any changes in BEA. One possibility is that the BEA measurements which reflect extracellular water content appear to be more variable and may not be accurate for identifying the fat-dominant later stage lymphoedema which is likely to occur in the current study [24].

Strengths of the trial is that it provides one of the few prospective datasets on the effect of weight loss on BCRL, and to the best of our knowledge the first to report effects of both home-based and supervised combined weight loss and resistance exercise programmes. Our study design included a run in period to allow participants to optimise standard lymphoedema management i.e. optimum skincare, arm mobility exercises, SLD, and use of compression hosiery particularly when exercising. Assessment of changes in body composition were undertaken using DXA. Also, changes of BCRL were assessed using both the perometer and BEA.

Limitations include the small sample size. Whilst this meets earlier cited conservative estimates [11, 12] we acknowledge these do not meet more recent larger recommendations [25]. The trial was of a short duration and achieved a relatively small degree of weight loss. It is important to note that the trial was not powered to detect an effect of weight loss and exercise on BCRL however trial data indicate possible intervention effects. Comparable reductions in BCRL between the combined weight loss and arm exercise and arm exercise only groups suggests the home-based exercise programme mediates much of the BCRL effect in the combined programmes [8]. However, an oversight in the protocol meant we did not collect data on actual adherence to the arm exercise programme at home which would inform whether resistance training was likely to be mediating the effects on BCRL.

The study was undertaken in women with long standing BCRL for whom weight loss may not be effective as chronic inflammation may lead to progressive and irreversible fibrosis of the lymphatic vessels. Combined weight loss and resistance exercise programmes may be effective for the prevention of BCRL in women with overweight and obesity. Particularly in combination with the use of arm compression sleeves, which are reported to be less effective in overweight women because compression garments are hard to fit and the fat component prevents effective compression and interferes with the lymphatic pump [26]. Weight control for the primary prevention of lymphoedema has not been studied, although a recent observational study (*N* = 1161) did not associate weight loss with protection against the development of BCRL [27]. Future studies could test different multi component weight loss interventions, for example a diet with a low inflammatory diet index [28], or including anti–inflammatory medications, or by achieving greater weight loss using a glucagon-like peptide-1 (GLP-1) and glucose-dependent insulinotropic polypeptide (GIP) receptor agonist [29].

## Conclusion

Women with BCRL and overweight and obesity will join and engage in diet and exercise weight loss programmes. BCRL remains a poorly managed condition with few effective management strategies [30]. The combined and independent effects of weight loss and exercise need further study both for the prevention and management of lymphoedema of different durations. Such studies require reliable measurement techniques to assess the effects of weight loss on RAVI and well-being.

### Supplementary Information

Below is the link to the electronic supplementary material.

Supplementary file1 (DOCX 16 KB)

Supplementary file2 (DOCX 21 KB)

Supplementary file3 (DOCX 18 KB)

## Data Availability

The datasets generated in the current study are available from the author on reasonable request.

## Abbreviations

ANCOVA: Analysis of covariance
BCRL: Breast cancer related lymphoedema
BEA: Bio impedance electrical analysis
DXA: Dual-energy X-ray absorptiometry
FACT: Functional Assessment of Cancer Therapy
FFM: Fat free mass
LOCF: Last observation carried forward
MFT: Manchester University Hospital Foundation NHS Trust
QOL: Quality of life
RAVI: Relative arm volume increase
TOI: Trial outcome indicator
