# Supplementary material for: BE-WEL trial (breast: evaluation of weight and exercise for lymphoedema) testing weight control and exercise programmes for women with breast cancer related lymphoedema: a feasibility trial
Source: Breast Cancer Res Treat. 2024 May 17;207(1):203–12. doi: 10.1007/s10549-024-07356-0 (PMC11230950; doi:10.1007/s10549-024-07356-0)
Supplement: Supplementary file 1 — Supplementary file1 (DOCX 16 KB) [file 10549_2024_7356_MOESM1_ESM.docx]

**Supplementary table 1: Inclusion and Exclusion criteria**

BE-WEL Trial (Breast: Evaluation of Weight and Exercise for Lymphoedema)

Testing weight control and exercise programmes for women with breast cancer related lymphoedema: a feasibility trial

Breast cancer research and treatment

Authors: Michelle Harvie, Karen Livingstone, Debbie McMullan, Mary Pegington, Cheryl Lombardelli, Judith Adams, Maggie Farragher, Emma Barrett, Nigel Bundred.

Corresponding Author: Michelle Harvie, Manchester University Hospital Foundation NHS Trust, Division of Cancer Sciences, Faculty of Biology, Medicine and Health, University of Manchester; michelle.harvie@manchester.ac.uk

| **Inclusion**  1. Women receiving maintenance therapy for breast cancer related arm lymphoedema i.e. compression sleeves + / - manual lymphatic drainage.  2. Overweight/obese BMI > 25 Kg /m^2^  3. Stable lymphoedema over the past 3 months defined as:   - No intensive therapy (i.e. no manual decongestive treatment). - No recorded 10% change in volume of the affected arm lasting > 7 days (assessed with perometer) - No lymphoedema related infections (cellulitis) requiring antibiotics.   4. Had lymphoedema sleeve re assessed by lymphoedema practitioner within the last 3 months  5. Any age.  6. Able to understand written instructions and record diet diaries.  7. No chemotherapy in previous 3 months or radiotherapy in the previous 12 months  8. Live within Greater Manchester/ Cheshire area to maximise uptake and retention to programme and study.  9. Sedentary < 40-minute moderate exercise / week  10. Willing to be randomised to the 4 treatment groups  11. Written informed consent. |
| --- |
| **Exclusion**  1. Metastatic disease or axillary recurrence  2. Physical/psychiatric condition which limits compliance to diet and exercise interventions  3. Eastern Cooperative Oncology Group (ECOG) performance status < 2  4. Already losing weight or following exercise programme.  5. Plans for surgery during the study period.  6. Pacemaker |
