# Supplementary material for: BE-WEL trial (breast: evaluation of weight and exercise for lymphoedema) testing weight control and exercise programmes for women with breast cancer related lymphoedema: a feasibility trial
Source: Breast Cancer Res Treat. 2024 May 17;207(1):203–12. doi: 10.1007/s10549-024-07356-0 (PMC11230950; doi:10.1007/s10549-024-07356-0)
Supplement: Supplementary file 2 — Supplementary file2 (DOCX 21 KB) [file 10549_2024_7356_MOESM2_ESM.docx]

Supplementary Table 2: Changes in self-reported dietary intake and physical activity between baseline and 12 weeks

BE-WEL Trial (Breast: Evaluation of Weight and Exercise for Lymphoedema) Testing weight control and exercise programmes for women with breast cancer related lymphoedema: a feasibility trial Breast cancer research and treatment

Authors: Michelle Harvie, Karen Livingstone, Debbie McMullan, Mary Pegington, Cheryl Lombardelli, Judith Adams, Maggie Farragher, Emma Barrett, Nigel Bundred.

Corresponding Author: Michelle Harvie, Manchester University Hospital Foundation NHS Trust, Division of Cancer Sciences, Faculty of Biology, Medicine and Health, University of Manchester; michelle.harvie@manchester.ac.uk

| \|  \|  \| Standard care  (n = 12)^a^ \| Supervised weight loss and exercise  (n =12) ^a^ \| Home-based weight loss and exercise  (n=16) ^a^ \| Home-based arm exercise only  (n = 17) ^a^ \| Supervised weight loss and exercise  vs standard care ^b^ \| Home-based weight loss and exercise  vs standard care ^b^ \| Home-based  arm exercise  vs standard care ^b^ \| \| --- \| --- \| --- \| --- \| --- \| --- \| --- \| --- \| --- \| \| Energy intake - kcal \| Baseline \| 1767  (1571-1962) \| 1666  (1445-1888) \| 1708  (1516-1891) \| 1855  (1631-2080) \|  \|  \|  \| \| Change over 12 weeks intervention \| --240  (-495 to +12) \| -243  (-478 to +8) \| -385  (-592 to -178) \| -50  (-156 to +54) \| -23  (-390 to + 343) \| -157  (-509 to +196) \| +206  (-132 to +545) \| \| Total fat -g \| Baseline \| 67  (57-76) \| 59  (47-71) \| 63  (54-72) \| 70  (56-84) \|  \|  \|  \| \| Change over 12 weeks intervention \| -10  (-23 to + 4) \| -10  (-22 to + 2) \| -17  (-26 to -8) \| +2  (-3 to + 7) \| -1  (-19 to + 16) \| -7  (-25 to + 10) \| 12  (-4 to +29) \| \| Saturated fat -g \| Baseline \| 23  (19 -27) \| 20  (15-26) \| 23  (18-28) \| 25  (18-31) \|  \|  \|  \| \| Change over 12 weeks intervention \| -3  (-9 to +3) \| -5  (-9 to 0) \| -7  (-11 to - 2) \| 1  (-2 to +3) \| -2  (-10 to + 5) \| -4  (-11 to +3) \| +4  (-3 to +11) \| \| Protein-g \| Baseline \| 74  (65-82) \| 77  (68-86) \| 71  (64-78) \| 79  (69-89) \|  \|  \|  \| \| Change over 12 weeks intervention \| -3  (-15 to +9) \| -8  (-16 to -1) \| -6  (-11 to -1) \| -4  (-9 to +1) \| -4  (-17 to +9) \| -3  (-13 to +7) \| -1  (-11 to +9) \| \| Carbohydrate -g \| Baseline \| 202  (165- 239) \| 209  (179-240) \| 200  (179-221) \| 221  (195-248) \|  \|  \|  \| \| Change over 12 weeks intervention \| -31  (-61 to – 1) \| -29  (-62 to +5) \| -47  (-75 to – 18) \| -10  (-24 to + 14) \| +5  (-42 to + 52) \| -16  (-61 to +28) \| +27  (-16 to +71) \| \| Moderate and vigorous activity - minutes / week \| Baseline \| 264  (76 - 695) \| 372  (66- 689) \| 302  (97-509) \| 454  (85- 524) \|  \|  \|  \| \| Change over 12 weeks intervention \| -194  (-370 to -17) \| -201  (-482 to –81) \| +16  (-207 to + 239) \| -166  (-428 to +95) \| +11  (-175 to 198) \| +175  (-24 to+340) \| +108  (-91 to +308) \|   ^a^ Mean (95% CI) for baseline and change from baseline to 12 weeks  ^b^ Mean (95% CI) change from baseline to 12 weeks versus change in standard care group. Standard care group N=12, Supervised weight loss and exercise group N=12, Home-based weight loss and exercise group N=14, Home-based arm exercise only group N=17  Last observation carried forward (LOCF) was used to impute missing data |
| --- | --- | --- | --- | --- | --- | --- | --- | --- | --- | --- | --- | --- | --- | --- | --- | --- | --- | --- | --- | --- | --- | --- | --- | --- | --- | --- | --- | --- | --- | --- | --- | --- | --- | --- | --- | --- | --- | --- | --- | --- | --- | --- | --- | --- | --- | --- | --- | --- | --- | --- | --- | --- | --- | --- | --- | --- | --- | --- | --- | --- | --- | --- | --- | --- | --- | --- | --- | --- | --- | --- | --- | --- | --- | --- | --- | --- | --- | --- | --- | --- | --- | --- | --- | --- | --- | --- | --- | --- | --- | --- | --- | --- | --- | --- | --- | --- | --- | --- | --- | --- | --- | --- | --- | --- | --- | --- | --- | --- | --- | --- | --- |
