# Supplementary material for: BE-WEL trial (breast: evaluation of weight and exercise for lymphoedema) testing weight control and exercise programmes for women with breast cancer related lymphoedema: a feasibility trial
Source: Breast Cancer Res Treat. 2024 May 17;207(1):203–12. doi: 10.1007/s10549-024-07356-0 (PMC11230950; doi:10.1007/s10549-024-07356-0)
Supplement: Supplementary file 3 — Supplementary file3 (DOCX 18 KB) [file 10549_2024_7356_MOESM3_ESM.docx]

**Supplementary Table 3: Changes in self- reported symptoms between baseline and 12 weeks** BE-WEL Trial (Breast: Evaluation of Weight and Exercise for Lymphoedema) Exercise for Lymphoedema) Testing weight control and exercise programmes for women with breast cancer related lymphoedema: a feasibility trial Breast cancer research and treatment

Authors: Michelle Harvie, Karen Livingstone, Debbie McMullan, Mary Pegington, Cheryl Lombardelli, Judith Adams, Maggie Farragher, Emma Barrett, Nigel Bundred.

Corresponding Author: Michelle Harvie, Manchester University Hospital Foundation NHS Trust, Division of Cancer Sciences, Faculty of Biology, Medicine and Health, University of Manchester; [michelle.harvie@manchester.ac.uk](mailto:michelle.harvie@manchester.ac.uk)

|  | **Change between baseline and end of the 12 week interventions ^a^** | | | | |
| --- | --- | --- | --- | --- | --- |
|  |  | **Standard care**  **(n = 12)** | **Supervised weight loss and exercise**  **(n =12)** | **Home-based weight loss and exercise**  **(n=16)** | **Home-based arm exercise only**  **(n = 17)** |
| **FACT -B TOI** | Baseline | 68.1  (35.6-103.7) | 73.3  (56.5-90.1) | 76.0  (50.4 -101.6) | 63.5  (30.3 -97.1) |
|  | Change over 12 weeks intervention | 4.7 (-0.7 to +10.2) | 3.2 (-1.7 to + 8.1) | 4.3 (-0.5 to +9.0) | 6.4 (0.6 to 12.2) |
| **FACT-F TOI** | Baseline | 79.0  (41.6- 116.4) | 91.8  (67.6-116) | 86.5  (51.5-121.5) | 78.5  (35.1-121.9) |
|  | Change over 12 weeks intervention | 7.5 (1.9 - 13.2) | -0.3 (-4.1 - +3.6) | 8.6 (2.4 - 14.8) | 8.1 (3.0 - 13.1) |
| **Arm sub-scale** | Baseline | 14.1  (5.3-22.9) | 15.4  (10.0 – 20.8) | 15.4  (9.0 – 21.8) | 11.8  (0.8 – 22.8) |
|  | Change over 12 weeks intervention | 0.6  (-0.6 to +1.7) | 0.0  (-2.1 to + 2.1) | -0.6  (-2.6 to +1.4) | 1.8  (-0.4 to +4.1) |
| **Self -rated arm swelling ^b^** | Baseline | 2.0  (1.3 – 2.8) | 2.1  (1.4-2.8) | 2.1  (1.3- 2.9) | 1.8  (1.0-2.6) |
|  | Change over 12 weeks intervention | 0.4  ( -0.2 to + 1.0) | 0.3  (-0.4 to +1.1) | -1.0  (-0.7 to +0.5) | -0.1  (-0.8 to +0.6) |

a Mean (95% CI) for baseline and change from baseline to 12 week using last observation carried forward value at 12 weeks

b Question B3 in the breast cancer specific sub-scale on the FACT questionnaire

FACT -B TOI Functional Assessment of Cancer Therapy-B Trial Outcome Index. FACT-F TOI Functional Assessment of Cancer Therapy-fatigue Trial Outcome Index
